# Possibility of Leishmaniasis Transmission in Jura, France

**DOI:** 10.3201/eid1806.120158

**Published:** 2012-06

**Authors:** Mohamed Kasbari, Christophe Ravel, Noel Harold, Bernard Pesson, Francis Schaffner, Jerome Depaquit

**Affiliations:** French Agency for Health and Safety Animal Health Laboratory, Maisons-Alfort, France (M. Kasbari);; University of Montpellier, Montpellier, France (M. Kasbari, C. Ravel);; University of Reims Champagne-Ardenne, Reims, France (M. Kasbari, B. Pesson, J. Depaquit);; French Institute for Public Health Surveillance, Saint-Maurice, France (N. Harold);; University of Strasbourg, Illkirch, France (B. Pesson);; University of Zurich, Zurich, Switzerland (F. Schaffner)

**Keywords:** leishmaniasis, transmission, sand flies, France, parasites, Phlebotomus perniciosus, canines

**To the Editor:** The report of a human cutaneous leishmaniasis case acquired in Clairvaux-les-lacs (*1*) led us to carry out an investigation with the veterinary clinics in Jura Department, France. Clairvaux-les-lacs is a lakeside resort located in Jura, one of the areas in France with the coldest average temperatures, and is clearly located outside the usual leishmaniasis-endemic area. At least 31 cases of canine leishmaniasis were diagnosed by veterinary clinics in Jura during 2007–2011. Because these dogs were native of or traveled in the leishmaniasis-endemic area along the Mediterranean Sea, all veterinarians considered the infections as acquired outside Jura.

Although phlebotomine sand flies have not been reported in Jura to date, *Phlebotomus perniciosus* sand flies, proven vectors of leishmaniasis, have been found in 2 areas neighboring Jura: Côte-d'Or and Saône-et-Loire (*2**,**3*). We have also recently caught *P. mascittii* sand flies, a species with an unknown vectorial competence, in the Swiss region of Jura, Alsace, Champagne-Ardennes, and Belgium. Therefore, the presence of sand flies in Jura, particularly in wet and milder microclimatic areas (as Clairvaux-les-lacs), is likely, and canine infections could have been acquired locally.

A recent model predicted that new at-risk areas are mostly located in western France along the Atlantic coast (*4*). In accordance with this model, we report new foci of autochthonous canine leishmaniasis in Deux Sèvres, Loire-Atlantique, and Loiret. Canine leishmaniasis cases contracted in the Rhine Valley in Germany (*5*) and the canine cases in Jura argue for a northeastern spread of the disease-endemic area along the Rhone-Rhine axis and mild microclimatic niches. Entomologic and serologic surveys will be carried out in summer 2012 in Jura to look for evidence of possible indigenous transmission of leishmaniasis. These data should supplement the current model of northern spread of leishmaniasis-endemic areas.
